# An illustrative case of endocardial fibroelastosis and recalcitrant intracardiac thrombosis: a case report

**DOI:** 10.1186/s12959-019-0199-3

**Published:** 2019-06-07

**Authors:** Denizhan Ozdemir, Isabel Oliva Cortopassi, Robert Lawrence McNamara

**Affiliations:** 1 Department of Internal Medicine, Yale New Haven Hospital, Yale School of Medicine, New Haven, CT USA; 2 Section of Radiology and Biomedical Imaging, Yale New Haven Hospital, Yale School of Medicine, New Haven, CT USA; 3 Section of Cardiovascular Medicine, Yale New Haven Hospital, Yale School of Medicine, Dana Clinic Building, 3rd Floor, 789 Howard Avenue, New Haven, CT 06519 USA

**Keywords:** Endocardial fibroelastosis, Atrial thrombus, Apixaban, Novel oral anticoagulant agent

## Abstract

**Background:**

Endocardial Fibroelastosis is diffuse, accentuated proliferation of ventricular endocardium causing a rare form of restrictive cardiomyopathy in both children and adults. It is an incompletely understood cause of heart failure predominantly in Sub-Saharan Africa associated with high morbidity and mortality. Atrial fibrillation and thrombus formation are common accompanying complications and portend a poor prognosis. Due to rarity of the condition in the developed countries and lack of evidence based options, the optimal strategy for anticoagulation is unclear.

**Case presentation:**

Herein, we describe a relatively asymptomatic patient with endocardial fibroelastosis who has been found to have atrial fibrillation and a large thrombus in the right atrium. Currently, there is no evidence-based strategy in the management of endocardial fibroelastosis-associated intracardiac thrombus. This case report illustrates a scenario by which the use of apixaban potentially benefited or prevented the thrombus formation compared with warfarin as demonstrated by imaging findings.

**Conclusions:**

The patients with endocardial fibroelastosis are at risk of developing intracardiac thrombus due to sticky substrate lining cardiac chambers while being relatively asymptomatic. No directed therapy is known for the management of heart failure and any complications of subsequent arrhythmias. The general recommendations follow those of same conditions in other hosts. Novel oral anticoagulant agents can be considered in the treatment of atrial thrombus in the appropriate settings.

## Background

Endocardial fibroelastosis (EFE) is defined as thickening of endocardial surface by proliferation of fibrous and elastic tissue [1, 2]. Although it mainly presents in pediatric population in Sub-Saharan Africa, EFE can also affect adults [3]. The etiology of EFE is unclear but proposed to be related to infectious agents and the climate due to variation with geographical distribution [4]. Histologically, the abnormal endocardium is composed of multiple layers of elastic and fibrous tissue that can functionally impair the contractility of cardiac chambers [5]. Due to underlying contractility dysfunction and propensity to coagulation in endocardial microenvironment, patients are susceptible to heart failure, atrial fibrillation and thrombotic complications [6–9]. Currently, the management follows the general rule of heart failure and atrial fibrillation from different etiologies [6–8].

## Case presentation

Herein, we describe a case report of EFE that developed atrial fibrillation and aggressive thrombosis of right atrium. A 68-year-old Nigerian man is brought into the emergency room with asymptomatic heart rate of 30 per minute, noted before a cardiopulmonary stress test which he was undergoing for evaluation of prognosis and candidacy for cardiac transplantation. His medical history is significant for endocardial fibroelastosis diagnosed 15 years ago, persistent atrial fibrillation with slow ventricular response, hypertension, moderate sleep apnea, pre-diabetes, and non-sustained ventricular tachycardia on ambulatory Holter monitoring for which he had previously declined an implantable cardioverter defibrillator (ICD). His medications included loop diuretics and warfarin. His social history is notable for immigration to the United States at mid-adult age and remote tobacco use. Family history is remarkable for multiple first-degree family members with sudden cardiac death before 50 years of age. He transferred his care to our health care system a few years prior to current presentation. Review of systems was notable for fatigue.

Physical exam demonstrated jugular venous distention with marked Y descent present, irregularly irregular heart rate, hepatomegaly, and ascites. LDL level was borderline elevated. Otherwise, laboratory values were all within normal limits, including hematologic evaluation, IgE levels and stool ova and parasites.

An electrocardiogram (EKG) revealed atrial fibrillation with slow ventricular response, rightward axis deviation, borderline low voltage in limb leads, and Q waves in lead III.

His first available transthoracic echocardiogram in our health system was performed in 2015. It revealed normal left ventricular cavity size, diastolic and systolic function with estimated ejection fraction of 55%; severely enlarged right atrium without thrombus; diffuse right ventricular endocardial thickening with normal systolic function and cavity obliteration; mild to moderate tricuspid regurgitation; and trace pericardial effusion. Cardiac catheterization outside this health care system reportedly showed restrictive pattern; however, details are not known. Initial cardiac magnetic resonance imaging (cMRI) in 2015 showed massive dilation of right atrium, distortion of right ventricle and mass-like thickening of mid-septum demonstrating diffuse and mildly heterogeneous enhancement on delayed post-contrast imaging with no evidence of intracardiac thrombus. From 2015 to 2017, the patient remained asymptomatic without hospitalization or any major illness and was able to work part-time. He was seen in scheduled intervals for the adjustments in his management and monitoring. His restrictive physiology and volume status were managed with low dose diuretics. He did not tolerate beta blocker due to bradycardia in the past. To reduce the risk of thromboembolic complications from atrial fibrillation, he was anticoagulated with apixaban for two years (2015 through 2017) until three weeks before presentation with no evidence of intracardiac thrombus on periodic cardiac MRI imaging. Three weeks prior to presentation, he was switched from apixaban to warfarin for unknown reasons.

**Fig. 1:**
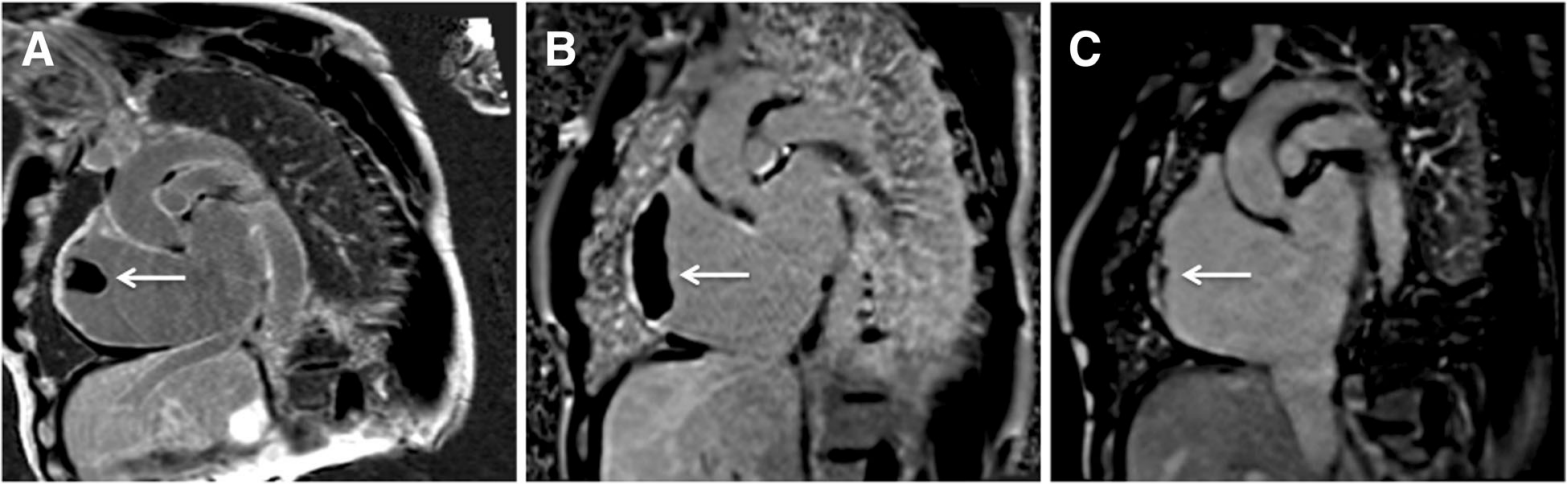
**a**, arrow: Short axis late gadolinium enhancement images through the right atrium demonstrate thrombus formation measuring 29 × 22 mm three weeks after switch from apixaban to warfarin. **b**, arrow: The thrombus enlarged to 71 × 21 mm 3 months after continuing use of warfarin. **c**, arrow: The thrombus diminished to 31 × 5 mm 5 months after discontinuation of warfarin and re-instating apixaban

During his hospitalization a cMRI was obtained to monitor any changes associated with EFE. It revealed a 29 × 22 mm thrombus in the right atrial cavity (Fig. 1a). There was no evidence of medication non-adherence, and his International Normalized Ratio (INR) was 2.6 at the time of detection of thrombus. He continued to take warfarin for anticoagulation to treat large thrombus in the right atrium for the next 3 months. Notably, his INR remained within the therapeutic range (2.0–3.0) nine out of eleven measurements over 3-month time period since thrombus was first detected, with only two sub-therapeutic levels of 1.7 and 1.8. However, the size of thrombus increased to 71 × 21 mm on the following repeat imaging (Fig. 1b). Due to progression of atrial thrombus, the apixaban was re-instated, and warfarin was discontinued. Repeat imaging 5 months after apixaban was restarted demonstrated the reduction in thrombus size to 31 × 5 mm (Fig. 1c). Subsequent imaging demonstrated further regression. From the first encounter the large thrombus was detected until the last clinic visit, he continued to remain asymptomatic and at his usual state of health without any evidence of thromboembolic or bleeding complications.

## Discussion and conclusions

Endocardial fibroelastosis (EFE) constitutes a type of restrictive cardiomyopathy predominantly seen in Sub-Saharan Africa [10]. The literature is scarce on EFE, and the etiology is not fully understood. However, infection, climate, and genetic factors are thought to be implicated [11, 12]. EFE is a distinct entity from endomyocardial fibrosis where hypereosinophilia is considered to be the original insult [13]. Also, it should not be confused with left ventricular non-compaction cardiomyopathy, where pronounced ventricular trabeculations are characteristic [14].

EFE manifests as excessive thickening of endocardium with subsequent myocardial dysfunction. The diagnosis is usually made with non-invasive testing including clinical features, cardiac imaging findings and exclusion of associated conditions such as hypereosinophilic syndrome, infections or storage diseases [15]. It often eventually leads to typical restrictive heart failure manifestations and cardiac arrhythmias, predominantly atrial fibrillation [7–9]. Due to altered endothelium lining associated with disorganized tissue growth, patients with EFE are also predisposed to thrombus formation [16, 17]. Unfortunately, no directed therapy for EFE is known because of rarity of the condition, and current recommended management follows those of heart failure in general such as addressing heart failure symptoms with optimization of volume status, managing rate control in case of atrial fibrillation with rapid ventricular rates, and risk stratification for conduction disturbances for candidacy of implantable devices among others [6, 18, 19]. Of particular note, recommendations for thromboembolic risk prevention and management of detected thrombus follow general guidelines as limited specific evidence exists in patients with EFE. In this case, no thrombus was noted on multiple cMRIs during treatment with a direct anticoagulant, apixaban, but a thrombus was noted and continued to enlarge on warfarin therapy despite therapeutic INR values. Subsequent switch back to apixaban demonstrated successful treatment. One explanation of the improved efficacy of the direct anticoagulant may be the predictable pattern of anticoagulant effect compared with the warfarin. In addition, the abnormal lining of endocardium in EFE may provide a particularly thrombogenetic surface, particularly in the hemodynamic setting of atrial fibrillation and high right atrial pressures due to restrictive heart disease. One recent case report is consistent with our findings. It discusses a potentially related scenario, a patient with left ventricular non-compaction cardiomyopathy who was successfully treated with a direct oral anticoagulant instead of warfarin [20]. Further studies may provide deeper understanding of thrombus formation and specific anticoagulation strategies in EFE.

## Abbreviations

cMRI: cardiac magnetic resonance imaging
EFE: Endocardial fibroelastosis
EKG: Electrocardiogram
ICD: Implantable cardioverter defibrillator
LDL: Low density lipoprotein
NOAC: Novel oral anticoagulant agent
